# Thymidine Kinase 2 Deficiency-Induced mtDNA Depletion in Mouse Liver Leads to Defect β-Oxidation

**DOI:** 10.1371/journal.pone.0058843

**Published:** 2013-03-07

**Authors:** Xiaoshan Zhou, Kristina Kannisto, Sophie Curbo, Ulrika von Döbeln, Kjell Hultenby, Sindra Isetun, Mats Gåfvels, Anna Karlsson

**Affiliations:** 1 Division of Clinical Microbiology F-68, Karolinska Institutet, Department of Laboratory Medicine, Karolinska University Hospital, Huddinge, Sweden; 2 Division of Clinical Chemistry, C1-72, Karolinska Institutet, Department of Laboratory Medicine, Karolinska University Hospital, Huddinge, Sweden; 3 Division of Metabolic Diseases, Karolinska Institutet, Department of Laboratory Medicine, Karolinska University Hospital, Huddinge, Sweden; 4 Division of Clinical Research Center, Karolinska Institutet, Department of Laboratory Medicine, Karolinska University Hospital, Huddinge, Sweden; 5 Division of Clinical Chemistry, Department of Laboratory Medicine, Lund University, Lund, Sweden; Vanderbilt University Medical Center, United States of America

## Abstract

Thymidine kinase 2 (TK2) deficiency in humans causes mitochondrial DNA (mtDNA) depletion syndrome. To study the molecular mechanisms underlying the disease and search for treatment options, we previously generated and described a TK2 deficient mouse strain (TK2^−/−^) that progressively loses its mtDNA. The TK2^−/−^ mouse model displays symptoms similar to humans harboring TK2 deficient infantile fatal encephalomyopathy. Here, we have studied the TK2^−/−^ mouse model to clarify the pathological role of progressive mtDNA depletion in liver for the severe outcome of TK2 deficiency. We observed that a gradual depletion of mtDNA in the liver of the TK2^−/−^ mice was accompanied by increasingly hypertrophic mitochondria and accumulation of fat vesicles in the liver cells. The levels of cholesterol and nonesterified fatty acids were elevated and there was accumulation of long chain acylcarnitines in plasma of the TK2^−/−^ mice. In mice with hepatic mtDNA levels below 20%, the blood sugar and the ketone levels dropped. These mice also exhibited reduced mitochondrial β-oxidation due to decreased transport of long chain acylcarnitines into the mitochondria. The gradual loss of mtDNA in the liver of the TK2^−/−^ mice causes impaired mitochondrial function that leads to defect β-oxidation and, as a result, insufficient production of ketone bodies and glucose. This study provides insight into the mechanism of encephalomyopathy caused by TK2 deficiency-induced mtDNA depletion that may be used to explore novel therapeutic strategies.

## Introduction

Mitochondrial DNA (mtDNA) depletion syndrome (MDS) is a group of rare and severe autosomal recessive disorders that are characterized by quantitative reduction of mtDNA in affected tissues [1]. MDS presents in early childhood or infancy with diverse symptoms such as progressive myopathy, hepatopathy and encephalopathy and is often fatal before the age of 3 years [2], [3]. The different clinical symptoms have been associated with different mutations in nuclear genes encoding proteins involved in the mtDNA metabolism [4]. Several mutations in the *thymidine kinase 2* (*TK2*), *deoxyguanosine kinase* (*DGUOK*), *polymerase gamma (POLG)* and *MpV17 mitochondrial inner membrane protein* (*MPV17)* genes have been linked to inheritable MDS in humans [5], [6], [7], [8]. Humans carrying TK2 deficiency primarily present with severe myopathy and display neurological phenotypes whereas DGUOK, POLG and MPV17 deficiencies have been associated with liver failure and encephalomyopathy [5], [6]. However, there are also reports of patients harbouring TK2 deficiency that have liver and brain involvement [9], [10], [11], [12], [13]. Patients with a certain TK2 mutation (R172W/R172W) have been shown to harbor TK2-associated mtDNA depletion in brain and liver in addition to muscle, confirming that TK2 mutations can cause multi-organ involvement [9]. The different phenotypes of TK2 and DGUOK are probably due to lack of enzymes with overlapping substrate specificities in the affected tissues or poor residual activity in tissues with high demand [14], [15], [16]. So far, there has been no human case of complete lack of functional TK2 protein, suggesting that residual TK2 activity might be required for human life. The phenotype in patients seems to correspond to the severity of the TK2 deficiency, where partial reductions of TK2 activity (14–45% of normal) lead to myopathy and severe reductions (<10% of normal) cause encephalomyophathy [9], [10], [17].

Currently there is no cure available for MDS and to increase the understanding of the mechanisms behind the syndrome we previously generated and described a mouse strain deficient in TK2 (TK2^−/−^) that we use as a model for human TK2 deficiency, and human TK2 deficient infantile fatal encephalomyopathy in particular [15]. Since the TK2^−/−^ mice completely lack TK2 activity and display multi-organ involvement, the model is similar to patients with TK2 mutations that involve brain and liver as well as muscle. The TK2^−/−^ mice were born with mtDNA depletion in adipose tissue, brain and skeletal muscle. Although the mice behaved and grew normally the first 7 days they demonstrated progressive loss of mtDNA content in several organs and at postnatal day 12 the mtDNA content was also decreased in liver. Although the liver structure was similar in the TK2^+/+^ and TK2^−/−^ mice, the latter showed an increased number of intracellular lipid vesicles. The TK2^−/−^ mice were also progressively hypothermic and displayed abnormal brown adipose tissue and an almost complete loss of hypodermal fat at 14 days of age when this fat layer is normally well developed. In addition, the adipocytes in the 14 days old TK2^−/−^ mice showed heterogeneity in size and accumulation of lipid vesicles in contrast to a homogeneous pattern seen in the TK2^+/+^ mice [15]. The attained data suggested an involvement of the lipid metabolism in the progressive loss of essential body functions in the TK2^−/−^ mice. Here we analysed the TK2^−/−^ mouse model to clarify the importance of mtDNA for liver function by studies of the lipid phenotype and functionality of the hepatic mitochondria as well as alterations in plasma. The obtained data show that the TK2-deficiency induced gradual loss of mtDNA in the liver leads to defect β-oxidation and, as a result, insufficient production of ketone bodies and glucose.

## Materials and Methods

### Animals

Official Swedish regulations for the use and care of laboratory animals were followed throughout and the experimental protocol was approved by the local ethical committee (Permit numbers: S104-09 and S135-11). TK2^+/−^ and TK2^−/−^ mice were developed and maintained as previously described [15]. F8 TK2^−/−^ mice were compared to TK2^+/+^ litter mates of the same generation, unless otherwise specified. Livers were immediately frozen in liquid nitrogen. Whole blood was collected through heart puncture and centrifuged at 800 g for 10 min. Serum and livers were stored at −80°C until use.

### Transmission Electron Microscopy

Electron images of mitochondria were obtained as previously described [18]. 40–50 nm ultra-thin sections from longitude parts were cut and examined in a Tecnai 10 transmission electron microscope (Fei, The Netherlands) at 80 kV. Digital images were randomly taken by using a Morada camera (Olympus Soft Imaging Solutions, Germany).

A stereological method was used to determine the volume density of the mitochondria in the cytoplasm of the hepatocytes. The areas of mitochondria profiles on printed images and the total area of the cytoplasm was measured by point counting using 2 cm^2^ lattices according to Weibel [19]. The ratio between the mitochondria areas and cytoplasm area was calculated. A pilot study was performed to determine the number of blocks and images needed for an appropriate sample using cumulative mean plot for evaluation [19]. Thus, 9 randomly taken images from two different sections were counted per animal (18 images/animal) and three different animals were used per group (54 images/group). A total of four groups were studied (7 and 12 days old TK2^+/+^ and TK2^−/−^). To determine the numbers of mitochondria per cell, estimations were done based on the two-dimensional structures in the images and the assumption that the size of mitochondria in the tissue correlates with the size of the area in the image.

### mRNA Expression

Total RNA from 7 and 12 days old mice (four of each genotype and age) was extracted by TRIzol® (Life Technologies) and quantified with the NanoDrop™ 2000 (Thermo Scientific). RNA quality assessment was performed using the Experion™ RNA StdSens analysis kit (Bio-Rad Laboratories). Oligo-dT-primed cDNA synthesis was performed with 1 µg total RNA using the High Capacity Reverse Transcriptase Kit (Applied Biosystems). The cDNA was diluted 1 to 10 before real-time PCR was performed on the Applied Biosystems PRISM 7000 using the SYBRgreen reagent (Applied Biosystems). Reactions were performed in triplicates and *hypoxanthine-guanine phosphoribosyltransferase (Hprt)* was selected as endogenous control. Data was collected on individual cDNA samples.

To identify additional genes involved in fatty acid oxidation that might be altered in TK2^−/−^ mice, a PCR screening array for 84 genes in the mouse fatty acid oxidation pathway (catalog no. PAMM-007, Qiagen) was conducted with liver tissue from 14 days old mice (three of each genotype). Total RNA was extracted using the RNA easy mini kit (Qiagen). The RNA integrity was assessed spectrophotometrically, and all samples had 260/280 ratios between 1.8–2.0 and 260/230 ratios above 1.7. Reverse transcription was done on 0.5 µg total RNA per sample using the RT2 First strand kit (Qiagen) according to the manufacturer’s protocol. SYBR green RT-PCR analysis was performed on an ABI 7500 fast cycler. The array evaluated the expression of 84 genes involved in fatty acid oxidation. The acquired data were analyzed for statistical differences using Qiagen’s software RT^2^ Profiler PCR Array Data Analysis version 3.5. Briefly the PCR array data were calculated by the comparative cycle threshold method, normalized against housekeeping genes, and expressed as mean fold change in TK2^−/−^ samples relative to TK2^+/+^ control samples. Genes of TK2^−/−^ mice that differed by >2-fold compared with TK2^+/+^ controls are included and highlighted in bold when p≤0.05.

### Protein Expression

Liver tissues from 7 and 12 days old mice (three of each genotype and age) were ground in liquid nitrogen, dissolved in 400 µl RIPA buffer, incubated on ice for two hours, centrifuged at 16000 g for 20 min at 4°C and the supernatants retrieved. Western blot analyses to determine adipophilin, cytochrome c oxidase subunit II (COX II) and voltage-dependent anion channel (VDAC) protein levels in liver protein extracts were performed as previously described [20]. Bio-RAD Quantity one software was used to determine the intensity of the bands. The expression of adipophilin and COXII was related to the expression of VDAC for each individual sample and the mean of the expression ratios were calculated. The adipophilin antibody was purchased from Progen biotechnik and the COX II antibody was a generous gift of professor Nils-Göran Larsson [20].

### Lipid Parameters in Liver

Liver tissues were extracted for lipids as described by Folch [21]. The delipidated liver tissues were dried and dissolved in 1 M NaOH for protein determination by Bio-Rad DC Protein Assay (Bio-Rad Laboratories). All hepatic lipids were related to the amount of protein in the liver tissues. For quantification of hepatic free and esterified cholesterol, d6-cholesterol was added as an internal standard to parts of the lipid extracts from 7 and 12 days old mice (four of each genotype and age). Cholesterol samples were hydrolyzed (for total cholesterol) and purified on ISOLUTE® MFC18 SPE columns (Biotage). The purified samples were trimethylsialylated and dissolved in hexane before analysis on GC-MS, and the amounts of cholesterol were quantified by isotope dilution mass spectrometry. Parts of extracts from 7 days old (eight TK2^+/+^ and nine TK2^−/−^), 10 days old (two of each genotype), and 12 days old (five of each genotype) mice were also used for quantification of hepatic triglyceride content using the TG GPO-PAP reagent (Roche). Samples were run in duplicates and the measurement was repeated once with new extracts. Absorption was measured at 492 nm (Infinite® F500 plate reader, Tecan) and analysed using the Magellan™ Data Analysis Software (Tecan).

### Serum Parameters

Size exclusion chromatography of plasma lipoproteins in samples from 7 and 12 days old mice (four of each genotype and age) was performed as described earlier [22] and their lipid content was measured online with cholesterol CHOD-PAP and TG GPO-PAP reagents (Roche). For quantitation of non-esterified fatty acids (NEFA), plasma was collected and immediately assayed according to the ACS-ACOD method by using the NEFA kit (WAKO, USA). Samples were run in duplicates or triplicates and the values compared to a standard curve performed on the same plate as the samples. Absorption was measured at 550 nm and the background at 750 nm (Infinite® F500 plate reader, Tecan).The assay was performed three times with samples from 12 days old mice (eight of each genotype), and twice for 7 days old mice (seven of each genotype).

Blood glucose levels were determined from whole blood by using the Accu-Chek (Roche). Blood ketone levels were determined in whole blood by using the Precision Exceed (Abbott). Six mice of each genotype were analyzed to determine the average ketone and glucose levels on postnatal day 14.

### Acylcarnitine Metabolites in Plasma

The determination of acylcarnitines was performed in 12 days old mice (three of each genotype) by a modification of previously described methods [23], [24] as follows:

*Sample preparation*: 50 µl plasma was diluted with a corresponding amount of isotope labelled internal standard mix (NSK-B, Cambridge Isotope Laboratories Inc.) and deproteinised with 500 µl acetonitril containing 0.3% v/v formic acid. The sample was vortexed vigorously and centrifuged. The supernatant was transferred to a clean vial and evaporated, and derivatised with 100 µl 3 M HCl in *n*-butanol (60°C, 15 min.). The sample was evaporated again and reconstituted with 100 µl methanol and 50 µl 10 mM formic acid.

*LC-MS/MS procedure:* 5 µl of the prepared sample was injected onto an Acquity UPLC™ BEH C18 2.1×50 mm, 1.7 µm chromatographic column (Waters). A gradient elution with water (mobile phase A) and methanol (mobile phase B), both containing 0.1% v/v formic acid, was set up to separate the analytes. The acylcarnitines in the resulting eluate were ionized in positive ion mode electrospray and determined with a tandem mass spectrometer (Xevo™ TQ MS, Waters) in MRM mode. Quantification of each compound was performed by internal standard methodology using the TargetLynx software (Waters). Plasma from 12 days old mice (three of each genotype) were analyzed in separate experiments.

### Betaoxidation in Liver

Mitochondrial β-oxidation was determined in liver homogenates according to a previously described method [25]. Briefly, 100–200 µg mitochondrial protein was used in each reaction. The conversion of [1-^14^C] palmitic acid (Sigma) into acid-soluble metabolites was studied in triplicate samples in the presence or absence of 50 µM antimycin A. The peroxisomal β-oxidation (antimycin A insensitive) was subtracted from the total β-oxidation. Livers of 14 days old mice (three of each genotype) were analyzed in separate experiments. Comparisons of mitochondrial β-oxidation were made within each run.

### ATP Production Rate and Citrate Synthase Activity in Liver

Mitochondria were isolated from liver tissue samples of 14 days old mice (three of each genotype). Mitochondrial ATP production rates (MAPR) and citrate synthase (CS) activity were determined as previously described [26]. Briefly, CS activity was measured at 35°C using a Spectronic GENESYS 10 UV-Vis spectrophotometer (Thermo Fisher Scientific). ATP production rates were determined at 25°C using a firefly luciferase and a Varioskan® Flash Spectral Scanning Multimode reader (Thermo electron corporation) in the presence of the following different substrate combinations: glutamate+malate (G+M), glutamate+succinate (G+S), pyruvate+malate (P+M), palmitoyl-l-carnitine+malate (PC+M), succinate (S) and succinate+rotenone (S+R). Activities were expressed as units per unit of CS activity in the mitochondrial suspension.

### Carnitine Palmitoyl Transferase (CPT) Activity in Liver

Total CPT activity was measured in isolated liver mitochondria from 14 days old mice (four of each genotype) according to a previously described method [27]. Briefly, each reaction contained 20 µg mitochondrial protein mixed in a 200 µl reaction buffer containing 20 mM HEPES, 1 mM EGTA, 220 mM sucrose, 40 mM KCl, 100 µM 5,5-dithio-bis(2-nitrobenzoic acid)(DTNB), 1.3 mg/ml BSA, and 40 µM palmitoyl-CoA, pH 7.4. The reaction was started by adding 1 mM carnitine (omitted in baseline) and monitored at 412 nm for 4 min at 25°C using a thermospecific spectrophotometer (Genesys 10 UV, VWR).

### Statistics

Two tailed, unpaired Student’s t-test was used to test for statistical significance of difference between TK2^+/+^ and TK2^−/−^ mice of the same age. Significance was set at p≤0.05.

## Results

### Altered Hepatic Mitochondria

**Figure 1:**
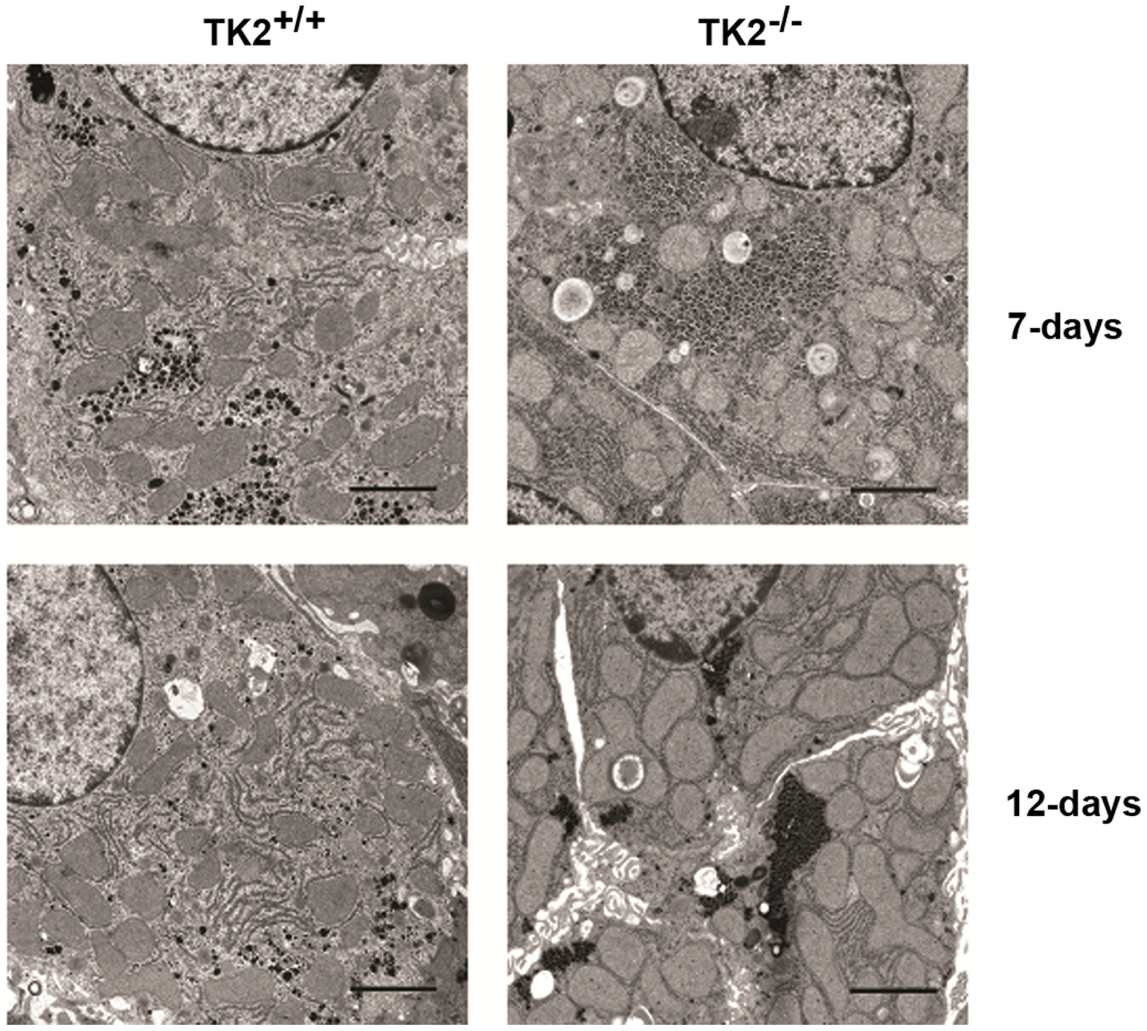
Transmission electron microscopy pictures of mitochondria in livers from TK2^+/+^ and TK2^−/−^ F6 mice. Bars = 2 µm. Sections shown are representative of the samples.

Transmission electron microscopy was performed on liver samples from the TK2^+/+^ and the TK2^−/−^ mice. Sections shown are representative of the samples (Fig.1). The volume of the mitochondria in TK2^−/−^ mice was larger compared to TK2^+/+^ mice of the same age. In 7 days old TK2^−/−^ mice, mitochondria constituted 24.0% of the total cell volume compared to 20.5% in TK2^+/+^ mice. An even larger difference was observed in 12 days old mice, where the mitochondria constituted 45.5% of the cell volume in TK2^−/−^ mice, compared to 27.8% in TK2^+/+^ mice (Table 1). To determine if the increased volume was due to hypertrophy or increased number of mitochondria the number of mitochondria profiles were estimated (2189 for TK2^+/+^ and 2128 for TK2^−/−^) and correlated to the total areas of the cytoplasm giving a mean number of mitochondria/µm^2^ cytoplasm of 0.52±0.02 for TK2^+/+^ and 0.54±0.08 for TK2^−/−^. However, the mean area/mitochondria was 0.540±0.010 µm^2^ for TK2^+/+^ and 0.866±0.046 µm^2^ for TK2^−/−^, all together showing that the mitochondria in the 12 days old TK2^−/−^ mice were hypertrophic.

**Table 1:** Relative volume of mitochondria in TK2^+/+^ and TK2^−/−^ mice.

Age (days)	Relative Volume (%)	
	TK2^+/+^	TK2^−/−^
7	20.5±3.2	24.0±1.5
12	27.8±0.2	45.5±4.5*

A stereological method was used to determine the volume density of the mitochondria in the cytoplasm from digital electron images taken by a Morada camera. Three different animals were analysed for each genotype and age. For each animal 9 randomly taken images from two different sections (18 images/animal) were counted making a total of 54 images/group. The P-value for statistical comparison (two-tailed unpaired Student’s t-test) between TK2^+/+^ and TK2^−/−^ of the same age is shown, *p≤0.05.

**Figure 2:**
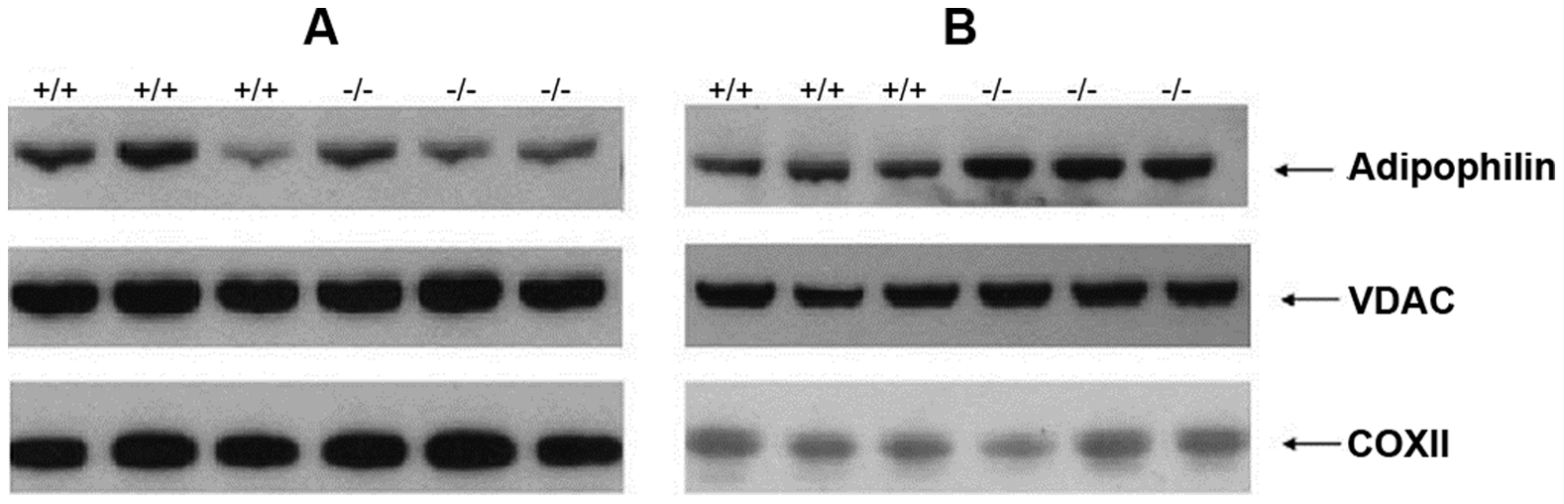
Hepatic expression of adipophilin, voltage dependent anion channel (VDAC) and cytochrome oxidase subunit II (COXII). Protein expression in livers of A) 7 days old and B) 12 days old TK2^+/+^ and TK2^−/−^ mice detected with Western blot. Protein samples are from three individuals of each genotype and age. Bio-RAD Quantity one software was used to determine the intensity of the bands and statistical comparisons (two-tailed unpaired Student’s t-test) between TK2^+/+^ and TK2^−/−^ were done. VDAC was used to normalize the data. Only 12 days old TK2^−/−^ mice exhibit 150% higher expression of adipophilin compared to TK2^+/+^ mice (p≤0.05), no other difference was detected.

It was previously shown that the mtDNA content in livers of 12 days old TK2^−/−^ mice constitutes approximately 70% compared to the TK2^+/+^ [15], [28]. To investigate whether the increased mitochondrial volume and mtDNA depletion in the TK2^−/−^ mice affected the protein expression in the hepatic mitochondria the expression levels of the mitochondrial protein cytochrome c oxidase subunit 2 (COXII) were measured in 7 and 12 days old mice. For both time points the COXII expression in the TK2^−/−^ mice was similar to the expression in the TK2^+/+^ mice (Fig. 2).

### Altered Hepatic Gene Expression and Lipid Content

**Figure 3:**
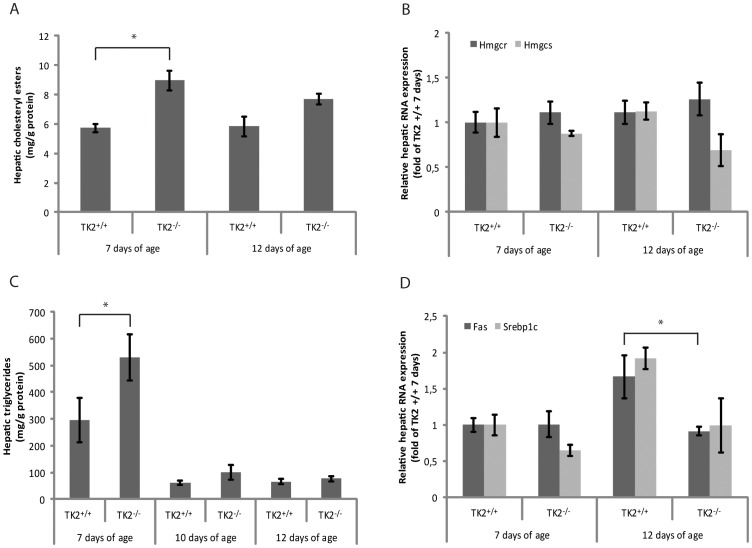
Hepatic gene expression and lipid content. A) Hepatic cholesteryl esters in 7 and 12 days old TK2^+/+^ and TK2^−/−^ mice determined with GC-MS. B) Relative hepatic mRNA expression of *3-hydroxy-3-methyl-glutaryl-Coenzyme A reductase* (*Hmgcr*) and *3-hydroxy-3-methyl-glutaryl-Coenzyme A synthase* (*Hmgcs*) in 7 and 12 days old TK2^+/+^ and TK2^−/−^ mice determined with real-time PCR. C) Hepatic triglycerides in 7, 10 and 12 days old TK2^+/+^ and TK2^−/−^ mice determined with an enzymatic assay. D) Relative hepatic mRNA expression of *fatty acid synthase* (*Fas*) and *sterol regulatory element binding protein 1c* (*Srebp1c*) in 7 and 12 days old TK2^+/+^ and TK2^−/−^ mice determined with real-time PCR. Data presented as mean ± SEM. Statistically significant difference (two-tailed unpaired Student’s t-test) compared to TK2^+/+^ of the same age, *p≤0.05.

The expression levels of adipophilin, a cellular indicator of the amount of stored lipids, were significantly increased in livers of 12 days old TK2^−/−^ mice (150%) compared to TK2^+/+^ mice (Fig. 2). Increased amounts of cholesteryl esters were detected in livers of 7 days old TK2^−/−^ mice compared to TK2^+/+^ mice of the same age. In livers of 12 days old mice no statistically significant difference was detected, although the mean value of the TK2^−/−^ mice was higher (p = 0.052) (Fig. 3A). The hepatic mRNA expression of *3-hydroxy-3-methyl-glutaryl-coenzyme A reductase* (*Hmgcr*), the rate limiting enzyme in cholesterol synthesis, was similar in both 7 and 12 days old TK2^−/−^ and TK2^+/+^ mice, but *3-hydroxy-3-methyl-glutaryl-coenzyme A synthase* (*Hmgcs*) *-1* displayed a trend towards decreasing expression levels with increasing age in the TK2^−/−^ mice (Fig. 3B). Hepatic triglyceride levels were elevated in 7 days old TK2^−/−^ mice compared to TK2^+/+^ mice of the same age, but no significant differences were observed between TK2^−/−^ and TK2^+/+^ mice of older age (Fig. 3C). The hepatic mRNA expression of *fatty acid synthase (Fas)*, a key enzyme in fatty acid synthesis, was decreased in the 12 days old TK2^−/−^ mice compared to TK2^+/+^ mice of the same age and the hepatic mRNA expression of the *sterol regulatory element binding protein 1c* (*Srebp1c*), which is responsible for the regulation of genes required for *de novo* lipogenesis, such as *Fas*, also displayed a similar expression pattern (Fig. 3D).

To further investigate the expression of genes involved in the fatty acid oxidation pathway in livers severely depleted of mtDNA, liver samples of 14 days old TK2^−/−^ mice containing <20% mtDNA, were analysed with PCR screening arrays and compared to TK2^+/+^. The expression levels of several genes were altered in the TK2^−/−^ mice (Table 2). However, only *Hmgcs-2* encoding the mitochondrial protein that catalyzes the first reaction of ketogenesis, was statistically significantly down regulated when analyzed using Qiagen’s software RT^2^ Profiler PCR Array Data Analysis.

**Table 2:** Genes differentially expressed in liver tissue of 14 days old TK2^+/+^ and TK2^−/−^ mice identified by a PCR-focused array.

Gene name	Fold change (TK2^−/−/^TK2^+/+^)
Acetyl-Coenzyme A acyltransferase 1A	−4.3
Acetyl-Coenzyme A acyltransferase 2(mitochondrial 3-oxoacyl-Coenzyme A thiolase)	−4.0
Acyl-Coenzyme A dehydrogenase family, member 10	−2.6
Acyl-Coenzyme A dehydrogenase family, member 9	−4.1
Acyl-Coenzyme A dehydrogenase, long-chain	−2.2
Acetyl-Coenzyme A acetyltransferase 1	−4.8
Acetyl-Coenzyme A acetyltransferase 2	−4.4
Acyl-CoA thioesterase 3	−7.5
Acyl-CoA synthetase long-chain family member 1	−4.0
Acyl-CoA synthetase long-chain family member 3	−4.5
Acyl-CoA synthetase long-chain family member 4	2.2
Acyl-CoA synthetase medium-chain family member 5	−6.8
3-hydroxybutyrate dehydrogenase, type 1	−34.8
3-hydroxybutyrate dehydrogenase, type 2	−7.4
Carnitine palmitoyltransferase 1a, liver	−2.9
Carnitine palmitoyltransferase 1b, muscle	2.2
Carnitine palmitoyltransferase 2	−3.1
Carnitine O-octanoyltransferase	−13.4
2.4-dienoyl CoA reductase 1, mitochondrial	−5.1
Fatty acid binding protein 1, liver	−2.7
Fatty acid binding protein 2, intestinal	−44.3
Fatty acid binding protein 3, muscle and heart	−3.5
Fatty acid binding protein 5, epidermal	−2.5
Fatty acid binding protein 6, ileal (gastrotropin)	−3.9
Glycerol kinase 2	−2.7
Glycerol phosphate dehydrogenase 2, mitochondrial	−4.4
Glycerol kinase	−3.3
Hydroxyacyl-Coenzyme A dehydrogenase/3-ketoacyl-CoenzymeA thiolase/enoyl-Coenzyme A hydratase(trifunctional protein), alpha subunit	−4.2
3-hydroxy-3-methylglutaryl-Coenzyme A lyase	−2.8
3-hydroxy-3-methylglutaryl-Coenzyme A synthase 1	−3.2
**3-hydroxy-3-methylglutaryl-Coenzyme A synthase 2**	−**3.7**
Lipase, hormone sensitive	−2.4
3-oxoacid CoA transferase 2A	−3.4
Peroxisomal trans-2-enoyl-CoA reductase	−5.4
Pyrophosphatase (inorganic) 1	−2.4
Protein kinase, AMP-activated, alpha 1 catalytic subunit	−2.7
Protein kinase, AMP-activated, beta 1 non-catalytic subunit	−2.2
Protein kinase, AMP-activated, beta 2 non-catalytic subunit	−2.7
Protein kinase, cAMP dependent, catalytic, alpha	−2.4
Protein kinase, cAMP dependent, catalytic, beta	−2.4
Protein kinase, AMP-activated, gamma 2 non-catalytic subunit	−2.2
Protein kinase, AMP-activated, gamma 3 non-catatlytic subunit	2.3
Solute carrier family 27 (fatty acid transporter), member 3	−4.6

Data are expressed as mean fold change in TK2^−/−^ samples relative to TK2^+/+^ control samples. The data analysis, including statistical comparisons, were done using Qiagen’s software RT^2^ Profiler PCR Array Data Analysis version 3.5. Genes of TK2^−/−^ mice (n = 3) that differed by >2-fold compared with TK2^+/+^ controls (n = 3) are included and highlighted in bold when p≤0.05.

### Elevated Levels of Cholesterol and NEFAs in Plasma

Plasma total cholesterol levels were elevated in both 7 and 12 days old TK2^−/−^ mice compared to TK2^+/+^ (Table 3). Separation of the lipoprotein fractions revealed that the cholesterol levels in both ApoA1-containing (HDL) and ApoB-containing (Chylomicron/VLDL/LDL) lipoprotein particles tended to be higher in the TK2^−/−^ mice of both ages, but the elevation was statistically significant only in 12 days old TK2^−/−^ mice. No changes in plasma triglyceride levels were detected between the genotypes (Table 3).

**Table 3:** Serum levels of cholesterol and triglycerides in ApoB-containing, ApoA1-containing and total lipoprotein particles, as well as total serum nonesterified fatty acids (NEFA).

Serumlipids	Genotype	TK2^+/+^		TK2^−/−^	
	Days of age	7	12	7	12
Cholesterol	ApoB-cont	1.05±0.10	1.12±0.05	1.31±0.17	1.32±0.05^*^
(mmol/l)	ApoA1-cont	1.92±0.04	2.39±0.02	2.03±0.09	2.58±0.04^**^
	Total	2.97±0.09	3.50±0.06	3.34±0.12^*^	3.90±0.06^**^
Triglycerides	ApoB-cont	0.84±0.02	0.69±0.09	0.89±0.04	0.83±0.11
(mmol/l)	ApoA1-cont	0.04±0.01	0.05±0.01	0.04±0.00	0.04±0.01
	Total	0.88±0.02	0.74±0.08	0.93±0.04	0.87±0.11
NEFA(mmol/l)	Total	0.79±0.10	0.60±0.02	0.91±0.08	0.76±0.02^**^

For each time point data from at least four different mice have been used (n≥4) and are presented as mean ± SEM. The P-value for statistical comparison (two-tailed unpaired Student’s t-test) between TK2^+/+^ and TK2^−/−^ of the same age group is shown, *p≤0.05, **p≤0.01.

The highly hydrophobic NEFAs are generally toxic to cells and therefore kept at low concentrations in plasma. The levels of NEFAs were similar in 7 days old TK2^−/−^ and TK2^+/+^ mice, but significantly increased in serum of 12 days old TK2^−/−^ mice (Table 3).

The blood glucose levels were determined in several mice. However, the blood sugar is increased as a response to food intake and all mice in this study were suckling pups with constant access to food, making it impossible to determine a fasting blood sugar value. Nevertheless, in some of the sacrificed 14 days old TK2^−/−^ mice blood sugar values as low as 2 mmol/l were observed. Such low values were not detected in the TK2^+/+^ mice. The ketone levels were also significantly reduced in 14 days old TK2^−/−^ mice, on average 1.6 mmol/l in TK2^−/−^ compared to 2.4 mmol/l in TK2^+/+^ mice.

### Accumulation of Acylcarnitines in Plasma

In mitochondrial fatty acid oxidation defects, metabolic blocks exist in the catabolic pathways of mitochondrial coenzyme A esters. Screening for disorders of fatty acid β-oxidation is commonly performed by determination of plasma acylcarnitine levels [29]. To determine if the plasma acylcarnitine metabolites differed between the mice the acylcarnitine profiles in plasma were determined by tandem mass spectrometry (Table 4). The TK2^−/−^ mice exhibited elevated levels of acyl- and 3-OH acylcarnitines with long chain lengths compared to the levels in TK2^+/+^ mice. In addition, the level of propionylcarnitine (C3) was significantly reduced and the levels of methylmalonylcarnitine (C4-DC) and buturylcarnitine (C4) were also decreased, although not significantly, in the TK2^−/−^ mice.

**Table 4:** Plasma acylcarnitine metabolites in 12 days old TK2^+/+^ and TK2^−/−^ mice.

Acylcarnitines	Carnitine	TK2^+/+^	TK2^−/−^	t-test
		[µM]	[µM]	p-value
Short-Chain				
	Acetyl (C2)	15.3±2.8	23.5±7.1	0.340
	**Propionyl (C3)**	**0.131±0.013**	**0.057±0.011**	**0.012**
	Butyryl (C4)	0.539±0.114	0.241±0.033	0.066
	Methylmalonoyl (C4-DC)	0.0257±0.0079	0.0133±0.0017	0.200
	Tiglyl (C5∶1)	0.0007±0.0007	0.0037±0.0023	0.284
	Isovaleryl (C5)	0.0410±0.0410	0.0317±0.0317	0.866
Medium-chain				
	Hexanoyl (C6)	0.0853±0.0354	0.0457±0.0122	0.349
	Octanoyl (C8)	0.0350±0.0055	0.0207±0.0003	0.060
	Decenoyl (C10∶1)	0.0013±0.0013	0.0017±0.0017	0.883
	Decanoyl (C10)	0.0470±0.0145	0.0243±0.0038	0.206
	Dodecenoyl (C12∶1)	0.0127±0.0009	0.0613±0.0473	0.362
	Lauryl (C12)	0.0840±0.0405	0.0683±0.0259	0.761
Long-chain				
	Myristoleyl (C14∶1)	0.0377±0.0182	0.0750±0.0285	0.331
	**Myristoyl (C14)**	**0.234±0.009**	**0.418±0.023**	**0.002**
	**3-OH myristoyl (C14-OH)**	**0.0090±0.0015**	**0.0163±0.0007**	**0.012**
	**Palmitoleyl (C16∶1)**	**0.034±0.010**	**0.115±0.008**	**0.004**
	3-OH palmitoleyl (C16∶1-OH)	0.0010±0.0010	0.0093±0.0047	0.156
	**Palmitoyl (C16)**	**0.524±0.016**	**1.433±0.106**	**0.001**
	**3-OH palmitoyl (C16-OH)**	**0.0030±0.0020**	**0.0193±0.0033**	**0.013**
	Octadecdienoyl (C18∶2)	0.0563±0.0274	0.1097±0.0452	0.370
	3-OH linoleyl (C18∶2-OH)	0	0.001±0.001	0.374
	**Octadecenoyl (C18∶1)**	**0.149±0.033**	**0.401±0.032**	**0.005**
	**3-OH oleyl (C18∶1-OH)**	**0.0017±0.0017**	**0.0123±0.0030**	**0.035**
	3-OH stearoyl (C18-OH)	0.0047±0.0047	0.0127±0.0127	0.585
	**Steaoryl (C18)**	**0.0567±0.0119**	**0.1267±0.0058**	**0.006**

For each time point data from three different mice have been used (n = 3) and are presented as mean ± SEM. Chains highlighted in bold are significantly different (comparisons were made with two-tailed unpaired Student’s t-test) between TK2^+/+^ and TK2^−/−^ mice and exhibit at least p≤0.05.

### Altered Hepatic Mitochondrial β-oxidation

**Figure 4:**
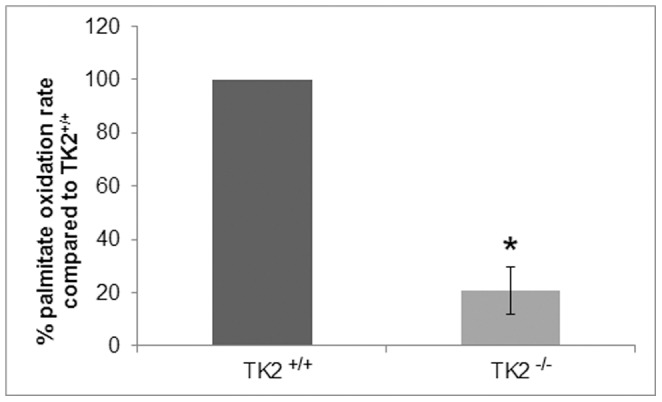
Mitochondrial palmitate oxidation rate in liver homogenates of 14 days old TK2^+/+^ and TK2^−/−^ mice. Three independent measurements were performed (TK2^+/+^ n = 3, TK2^−/−^ n = 3) and comparisons between the genotypes were made within each measurement. Data presented as per cent activity (mean ± SEM) compared to TK2^+/+^. The P-value for statistical comparison (two-tailed unpaired Student’s t-test) between TK2^+/+^ and TK2^−/−^ is shown, *p≤0.05.

The accumulated data pointed to impaired β-oxidation and for that reason the fatty acid oxidation was determined in liver homogenates from 14 days old mice (Fig. 4). The TK2^−/−^ mice demonstrated considerably reduced mitochondrial palmitate oxidation rate within each measurement, exhibiting only 9–40% of the oxidation rate observed in the TK2^+/+^ mice.

### Normal MAPR

It is known that mtDNA depletion may interfere with respiratory chain ATP production [27]. In 14 days old TK2^−/−^mice the mtDNA levels had drastically dropped below 20% compared to TK2^+/+^, however, the mtDNA depletion did not affect the MAPR at this point (Table 5).

**Table 5:** Mitochondrial ATP production rates standardized with citrate synthase activity.

genotype	Substrate combination (units/unit CS)					
	G+M	G+S	PC+M	P+M	S	S+R
TK2^+/+^	1.94±0.83	3.81±2.28	1.38±0.54	3.38±1.79	3.63±2.48	1.53±0.2
TK2^−/−^	1.69±1.03	2.86±1.50	1.13±0.51	2.27±1.31	1.87±1.54	1.38±1.25

CS: citrate synthase; G: glutamate; M: malate; S: succinate; P: pyruvate; PC: palmitoyl-L-carnitine; R: rotenone. Mitochondrial CS activity was similar in the genotypes. For each time point data from 14 days old mice, three from each genotype, have been used (n = 3) and are presented as mean ± SEM. Statistical comparisons (two-tailed unpaired Student’s t-test) between TK2^+/+^ and TK2^−/−^ were made but no statistical differences were detected.

### Altered CPT Activity

**Figure 5:**
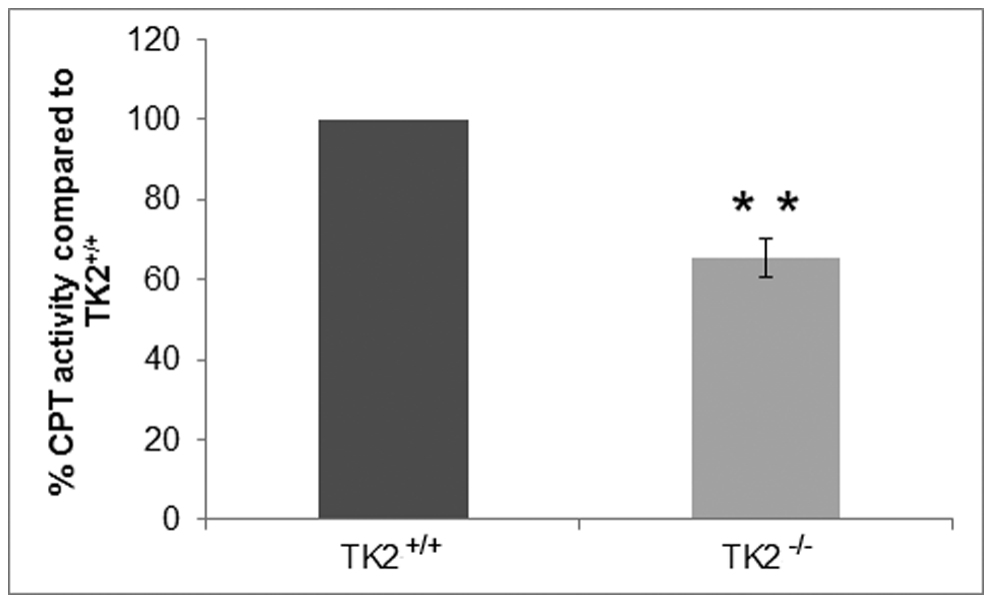
Carnitine palmitoyltransferase activity in hepatic mitochondria from TK2^+/+^ and TK2^−/−^ mice 14 days of age. The results represent data from four individuals of each genotype. Data presented as per cent activity (mean ± SEM) compared to TK2^+/+^. The P-value for statistical comparison (two-tailed unpaired Student’s t-test) between TK2^+/+^ and TK2^−/−^ is shown, **p≤0.01.

The observed MAPR in 14 days old mice was essentially normal also when the substrates entered through fatty acid oxidation (palmitoyl-L-carnitine plus malate). A key regulatory site controlling the flux of long-chain fatty acids through β-oxidation is the mitochondrial carnitine system that catalyzes the transport of long-chain fatty acids into the mitochondrial matrix. Since the gene expression level of *carnitine palmitoyltransferase 1A (CPT1A)*, which is responsible for the formation of long-chain acylcarnitines from activated fatty acids and free carnitine, was decreased (Table 2), the CPT activity in the livers of 14 days old mice was determined (Fig. 5). The method used determined the activity of both carnitine palmitoyltransferases, CPT1A and CPT2. The livers of TK2^−/−^ mice displayed approximately 65% CPT activity compared to TK2^+/+^ mice.

## Discussion

In this study liver and plasma from both symptom-free (7 days old) and symptomatic (12 and 14 days old) TK2^−/−^ mice were used to clarify how hepatic mitochondria are involved in the development of the encephalopathic phenotype of the TK2^−/−^ mice. The hepatic cells of TK2^−/−^ mice progressively lose the mtDNA and contain approximately 90% at postnatal day 7, 70% at postnatal day 12 and less than 20% at postnatal day 14, compared to the TK2^+/+^ mice. The TK2^−/−^ mice retained normal expression of the mtDNA encoded protein COXII in the hepatic cells both at day 7 and 12, showing that they had enough mtDNA to maintain normal protein levels. It has been shown before that partially mtDNA depleted cells can retain COXII protein levels [30], [31]. In a study of brain and heart of 13 days old TK2 knock-in mice several different mitochondrial initiation transcription factors were unaltered although the mtDNA content was reduced [28].

The loss of mtDNA content in hepatocytes was accompanied by an increase in mitochondrial volume, which was almost doubled in the TK2^−/−^ mice at day 12. Mitochondrial volume homeostasis is a housekeeping function that is essential to maintain the structural integrity of the mitochondrion. Increased matrix volume has also been suggested to activate the respiratory chain and thereby increase ATP production [32], [33]. The identified increase in matrix volume could, thus, be interpreted as a response to energy depletion. Furthermore, microscopy data showed altered mitochondrial structure and accumulation of lipid vesicles in the liver cells of both 7 and 12 days old TK2^−/−^ mice. In addition, hepatic adipophilin protein expression, a cellular indicator of stored lipids, was significantly increased in 12 days old TK2^−/−^ mice. Macrovesicular steatosis has also been seen periportally in liver of human patients harboring the multi-organ involvement form of TK2 mutations in which mtDNA depletion has been detected in brain and liver as well as muscle [9]. However, measurements of hepatic cholesteryl esters and triglycerides in mice older than 10 days did not show any significant alterations between TK2^−/−^ and TK2^+/+^, suggesting an alteration in the components of lipid vesicles in these mice which needs further investigation.

Lipids, such as cholesterol and NEFAs were elevated in 12 days old TK2−/− mice. The increased blood lipids may stem from a number of factors, such as intake from food and hepatic and peripheral clearance from and output to the bloodstream. However, high levels of circulating lipids may be an indicator of fat being released from the adipose tissue, contributing to the accumulation of lipid droplets in the liver cells of the TK2^−/−^ mice. Consistent with an altered lipid metabolism in the TK2^−/−^ mice abnormal adipose tissue development together with a moderate impairment of mitochondrial respiratory function was also found in TK2 knock-in mice [34]. Increased NEFAs accompanied by an exponential increase in total ketone bodies in plasma are also seen in patients suffering from starvation. However, the gastrointestinal tracts of the sacrificed TK2^−/−^ mice were filled with milk and the ketone-bodies were decreased in the TK2^−/−^ mice, not reflecting a typical profile for starvation. It is possible that hormonal imbalances e.g. caused by loss of pancreatic functions are also contributing to the deteriorating condition. However, our own preliminary investigations of insulin levels in plasma have not yet indicated any difference between the TK2^−/−^ and TK2^+/+^ mice, although it should be mentioned that these measurements were performed on non-fasted mice (data not shown).

Since the accumulated data suggested a defect in the fatty acid oxidation in the TK2^−/−^ mice plasma of 12 days old mice was screened for fatty acid oxidation disorders. Long-chain acylcarnitines and 3-OH acylcarnitines were found to accumulate in TK2^−/−^ mice suggesting an impaired β-oxidation. Therefore the palmitate oxidation rate was analyzed in liver mitochondria and found to be decreased by 60–90% in the TK2^−/−^ mice. However, due to limited sample size and inability to fast the mice the absolute palmitate oxidation rates could not be determined.

The severe drop in mtDNA levels in the TK2^−/−^ mice occurred rapidly between postnatal day 12 and 14, and it is likely that residual activities together with activities of newly synthesized mtDNA encoded proteins, were enough to maintain the respiratory chain activity at this point. The increased mitochondrial volume in the TK2^−/−^ liver cells may also contribute to further activate the respiratory chain. Other compensatory mechanisms such as increased mitochondrial transcript or protein half-lives, which have been suggested to occur in muscle tissue harboring severe mtDNA depletion due to TK2 deficiency [31], could also be involved. The observed MAPR was normal also for substrates entering through the fatty acid oxidation as palmitoyl-L-carnitine plus malate, suggesting that the alteration in the β-oxidation would be prior to the formation of acylcarnitines from fatty acids and carnitine. In the analysis of genes involved in the fatty acid oxidation pathway the expression of carnitine palmitoyltransferase *CPT1A,* which is responsible for conjugation of carnitine to long chain acyl-CoAs, was reduced. To test if the CPT1A activity was altered in 14 days old TK2^−/−^ mice a method determining total CPT activity was used and the activity was found to be reduced to 65% compared to TK2^+/+^. Since CPT1A is the overall rate-limiting step in the β-oxidation of long chain fatty acids it is likely that the reduced CPT1A activity is participating in the altered β-oxidation in the TK2^−/−^ mice. CPT1A is regulated both by malony-CoA, which inhibits its activity, and by the curvature and lipid characteristics of the outer mitochondrial membrane [35]. The methylmalonyl-CoA levels were unaffected in the TK2^−/−^ mice but the lipid profile of the TK2^−/−^ mice was altered and it is possible that it contributes to a decrease in CPT activity.

In 12 days old TK2^−/−^ mice the fatty acid oxidation was impaired but the observed ketone and glucose levels were normal. However, in 14 days old TK2^−/−^ mice the β-oxidation was reduced in the liver and the observed ketone and glucose levels were dramatically decreased. Since the brain cannot utilize fatty acids directly it is dependent on the liver to produce ketone bodies through fatty acid β-oxidation. These mechanisms are of vital importance for normal brain function when the organism is on a carbohydrate restricted diet. The brain has high ATP requirement and low regenerative capacity and is highly sensitive to mtDNA depletion. We have previously shown that lack of TK2 activity causes a decline in mtDNA levels in neurons *in vivo* [36]. The depletion of mtDNA caused impaired mitochondrial bioenergetic function and degeneration of selected neuronal types. The symptoms in patients with TK2 mutations that cause mtDNA depletion in brain and liver are similar to what is observed in the TK2^−/−^ mouse model. Besides the major reduction of mtDNA in muscle, liver and brain they also show an early disease onset, accumulation of fat in the liver in form of macrovesicular steatosis, and seizures as a late manifestation similar to the mice [9]. However, the TK2^−/−^ mouse completely lacks TK2 activity and displays more severe neurological features compared to patients with TK2 mutations [36]. These differences may reflect different tissue-specific dNTP pool regulatory pathways between the species. It has also been suggested that the mouse brain is more dependent on the mitochondrial nucleotide salvage pathway than other tissues. The obtained data of the TK2^−/−^ mice resembles symptoms seen in patients with progressive liver failure due to DGUOK deficiency that commonly present with hypoketotic hypoglycemia in combination with lactic acidosis and mild neurological involvement [37]. These patients have profound mtDNA depletions in liver which has been observed already at the prenatal stage in one patient [37], [38]. The cause of hypoglycemia in these patients has been suggested to be due to an islet cell hyperplasia causing hyperinsulinism [37]. A patient with a MPV17 mutation experienced improved liver function upon continuous glucose infusions and regular feeding [39]. Even though reduced β-oxidation or altered ketogenesis have not been described in patients harbouring TK2 deficiency with liver and brain involvement it might not have been studied and warrants further attention in this patient group. Altered ketogenesis has previously been described in patients with other hepatocerebral mtDNA depletion syndromes and it would be relevant to investigate if these patients suffer from reduced β-oxidation or CPT-activity as well.

The mtDNA content in the brain of the TK2^−/−^ mice was lower already at birth, and was somewhat further decreased at 14 days of age. However, the TK2^−/−^ mice were born with normal levels of mtDNA in the liver and developed severe mtDNA depletion by postnatal day 14 (<20% of normal mice). The severe mtDNA depletion coincided with severely reduced mitochondrial β-oxidation in the liver most likely due to loss of CPT1A activity. We propose that the gradually increasing failure of the liver to produce ketone bodies and glucose for the brain diminishes the remaining brain function and leads to loss of consciousness and ultimately death of the TK2^−/−^ mice. A better understanding of the primary and secondary factors that influence TK2 progression, and liver damage due to mtDNA depletion, may contribute to better prognosis and adequate treatments. In the absence of a cure for MDS the achieved results provide additional insight in the pathogenesis that can be used to explore novel therapeutic strategies.
